# A Study on the Development of a Korean Metabolic Syndrome Questionnaire Using Blood Stasis Clinical Data

**DOI:** 10.1155/2019/8761417

**Published:** 2019-05-27

**Authors:** Byoung-Kab Kang, Soobin Jang, Mi Mi Ko, Jeeyoun Jung

**Affiliations:** Clinical Medicine Division, Korea Institute of Oriental Medicine, Daejeon, Republic of Korea

## Abstract

**Objective:**

The aims of this study were to extract clinical indicators related to metabolic diseases using the Blood Stasis Questionnaires I and II (BSQ-I and II) developed in 2013 and 2014, respectively, and to develop a BSQ on metabolic syndrome (BSQ-MS).

**Methods:**

A total of 2,158 patients, comprising 1,214 from 7 traditional Korean medical hospitals in 2013 and 944 from 3 traditional Korean medical hospitals in 2014, were asked to complete the BSQ-I and BSQ-II. For the 370 patients who met the metabolic syndrome criteria, reliability and validity of the BSQ-MS were assessed using Cronbach's alpha, while prediction accuracy was determined by logistic regression.

**Results:**

The BSQ-MS included a total of 15 clinical signs and symptoms. It showed satisfactory internal consistency (Cronbach's *α* coefficient=0.70) and validity, with significant differences in mean scores between the blood stasis (14.09±6.14) and non-blood stasis (9.09±5.60) subject groups. The cut-off value of BSQ-MS score was 9 points, the area under the receiver operating characteristic curve was approximately 77%, the sensitivity and specificity of the diagnostic accuracy according to the cut-off value were 82.9% and 49.7%, respectively, and the sensitivity and specificity of the prediction accuracy by logistic regression were 72.2% and 71.6%, respectively.

**Conclusion:**

These results suggest that the BSQ-MS is an appropriate instrument for estimating blood stasis in patients with metabolic syndrome, although its sensitivity for diagnosis according to the cut-off value is low.

## 1. Introduction

In Oriental Medicine, blood stasis is a disease symptom in which blood becomes stagnant in certain parts of the body. Blood stasis is an old diagnostic concept dating back to the Huangdi Neijing and has been continuously developed in terms of medical diagnosis, pathology, and treatment [1]. While many efforts have been made to develop questionnaires for diagnostic standardization of blood stasis, [2] as well as basic research centering around Korean, Chinese, and Japanese literature for the development of a gynecological disease questionnaire [3], there is no standard diagnostic tool available in Korea. A blood stasis symptom questionnaire was developed by Yang et al. [4], followed by the publication of the revised questionnaire after confirmation of its reliability and validity [5]; however, it is not actively used in clinical practice.

The Korea Institute of Oriental Medicine combined the blood stasis diagnostic questionnaires developed in Korea, China, and Japan [4, 6, 7]. Due to the systematic differences in traditional medicine between Korea, China, and Japan, diagnostic standardization related to blood stasis has not been established. Since there is a combination of similar and different concepts, items were combined or separated by a panel of experts. Among the 36 items that were agreed upon after removal of duplicates, 3 women-related items were further excluded to result in 33 items for the development of the Blood Stasis Questionnaire I (BSQ-I). The reliability and validity of the BSQ-I was assessed using 1,214 subjects recruited from 6 oriental hospitals in 2013 [8]. Furthermore, the reliability and validity of the BSQ-II, which was expanded to include additional items from blood stasis research, were tested on 942 subjects recruited from gynecology, cardiovascular, and musculoskeletal areas in 2014 [9].

Metabolic syndrome is a morbid condition, which is manifested by central obesity, abnormal glucose tolerance, lipodystrophy, and hypertension. Oriental Medicine clarified that obesity is classified as phlegm-dampness. It is often accompanied with qi stagnation and blood stasis [10]. According to the principle, metabolic syndrome was defined as criteria of waist circumference (men > 102 cm; women > 88 cm); triglycerides ≥ 150 mg/dL; HDL cholesterol (men < 40 mg/dL; women < 50 mg/dL); blood pressure ≥130/≥85 mmHg; and fasting glucose ≥ 110 mg/dL. When 3 of 5 of the listed characteristics are present, a diagnosis of metabolic syndrome can be made.

The BSQ developed by the Korea Institute of Oriental Medicine is a questionnaire used for the diagnosis of blood stasis regardless of the disease. There is currently a lack of an oriental clinical index or questionnaire for the diagnosis of blood stasis with regard to metabolic syndrome. In the present study, we aimed to analyze clinical data collected from the developmental research of the previous BSQ in order to extract the main clinical indicators related to metabolic syndrome and apply these as the basis for the development of a BSQ for metabolic syndrome (BSQ-MS).

## 2. Research Methods

### 2.1. Research Design and Data Collection

This study was a multisite clinical study with a cross-sectional observational design. A total of 2,156 subjects were recruited from 8 domestic oriental hospitals between May 2013 and November 2014. For each subject, consent was initially obtained. Clinical data were collected by classifying each subject into either blood stasis (BSS) or non-blood stasis (Non-BSS) groups using a simple survey in order to homogenize the distribution of BSS and non-BSS by age and gender in each hospital. Blood stasis was diagnosed by two experts. To minimize the differences in blood stasis diagnosis between the two expert physicians, they each independently evaluated the subject at the same time. The physicians had graduated from the College of Oriental Medicine (6 years) and had at least 3 years of clinical experience. They received standard operating procedure training on blood stasis diagnosis and conducted the diagnosis according to standard operating procedure guidelines. This study was conducted after receiving approval from the Institutional Review Board from each participating college and the Korea Institute of Oriental Medicine (IRB No. I-1310/001-001-03).

### 2.2. Inclusion and Exclusion Criteria

Inclusion and exclusion criteria were as follows.

*Inclusion Criteria*

1. Age between 25 and 65 years.
2. Voluntarily signing the clinical research consent form, or legal guardian consent if they could not consent with free will.
3. Trust in the researcher, and willingness to cooperate and abide by the limitations throughout the duration of the study.
4. Consent to having blood drawn and the purpose of the study.

*Exclusion Criteria*

1. Mental disorder with communication difficulties.
2. Lack of consciousness, critical illness, or communication difficulties.
3. Pregnancy.
4. Presence of diseases that can affect the evaluation of the research other than the abovementioned criteria at the discretion of the researcher.

### 2.3. Categorization of Metabolic Syndrome

This study used the diagnostic criteria suggested by the National Cholesterol Education Program (NCEP) of the United States. The criteria were as follows.

- Abdominal obesity: waist circumference of 102 cm for males (90 cm for Asians) or 88 cm for females (85 cm for Asians) or higher.
- Triglycerides (TG): 150 mg/dl or higher.
- HDL cholesterol: 40 mg/dl for males and 50 mg/dl or lower for females.
- Fasting glucose: 100 mg/dl or higher or currently being treated for diabetes.
- Blood pressure: 130 mmHg or more systolic, or 85 mmHg or more diastolic.

Generally, metabolic syndrome is diagnosed if an individual meets 3 of the 5 criteria. However, in the present study, information about abdominal obesity was missing from the collected clinical data. Therefore, subjects were categorized with metabolic syndrome if they met 3 of the 4 criteria.

### 2.4. Clinical Indicators of Metabolic Disease Blood Stasis

The diagnostic criteria for clinical indicators of metabolic disease in China proposed by Fu et al. (2012) are as follows.

 Clinical index: 3 points

1. Angina pectoris.
2. Chest pain without angina pectoris.
3. Blackish red tongue.
4. Ecchymosis of tongue.
5. Stabbing pain.

Clinical index: 2 points

1. Pain at night.
2. Blackish red lips.
3. Sublingual varicosities.
4. Rough pulse^‡(-)^.
5. Cheek pain^*∗*(+)^.
6. Blackish red gingiva^*∗*(+)^.
7. Dark purple of palate mucosa^*∗*(+)^.
8. Easy bruising^*∗*(+)^.

Clinical index: 1 point

1. Scaly and rough skin.
2. Dark coloration of periocular region.
3. A dark coloration of the face.
4. Cyanosis^‡(-)^.

^‡^ means excluded index and *∗* means added index.

 The clinical indicators in the BSQs developed by the Korea Institute of Oriental Medicine comprise 5-point scales (1=false', ‘2=a little true', ‘3=average', ‘4=severe', ‘5=very severe'), except for the dichotomous “angina pectoris.” For each clinical index with a level higher than ‘a little true (2),' 3, 2, or 1 point(s) assigned to each index in Fu et al. [11] was assigned in the same manner. If the clinical index was ‘false (1),' 0 points were assigned. For the dichotomous clinical index of ‘having angina pectoris,' 3 points were given if the symptom existed and 0 points were assigned if the symptom did not exist. Among the 1-point clinical indicators of Fu et al. [11], “cyanosis” was excluded because it was not included in the BSQ. Among the 2-point clinical indicators, ‘rough pulse' was excluded due to its low relevance to metabolic disease. However, all the 11 clinical indicators suggested by Fu et al., except “cyanosis”, were judged to have high relevance to metabolic disease through 2 rounds of expert Delphi method [12]. Four additionally relevant clinical indicators, namely, ‘cheek pain,' ‘blackish red gingiva,' ‘dark purple of palate mucosa,' and ‘easy bruising,' were added to the 2-point clinical indicators. Using a total of 15 clinical indicators, the metabolic disease blood stasis score was calculated. The maximum value of the total score was 32 points.

### 2.5. Statistical Analysis

**Figure 1:**
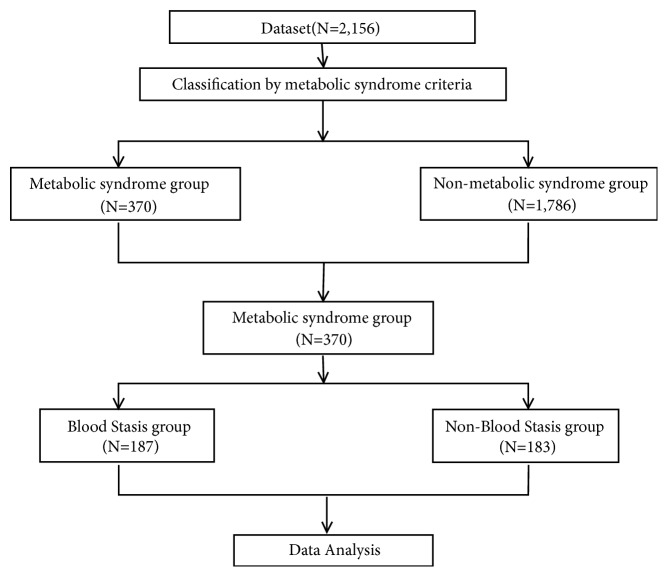
Participant selection flowchart.

Only 370 subjects categorized with metabolic syndrome out of a total of 2,158 were analyzed (Figure 1). Continuous variables were recorded as the mean ± standard deviation, while categorical variables were recorded as frequency (percentage). An independent two-sample t-test was used to compare the total metabolic disease blood stasis score calculated as the total of 15 clinical indicators for the existence of metabolic syndrome blood stasis. We performed a logistic regression to evaluate the importance of each clinical index. For all analyses, p-values smaller than the significance level of 0.05 were considered statistically significant. All analysis results were obtained with SAS 9.4 (SAS Institute, Cary, NC, USA).

## 3. Results

### 3.1. General Subject Characteristics

From a total of 2,156 subjects, 370 subjects were categorized with metabolic syndrome. These included 187 subjects in the BSS group and 183 subjects in the non-BSS group. The proportion of males with metabolic syndrome (208 subjects; 56.22%) was approximately 4% higher than that of females, but this was not statically significant. The proportion of males in BSS and on-BSS groups was higher than that of females, but this was not statically significant. The age of the non-BSS group was statistically significantly higher than that of BSS group (p=0.0313). Although systolic blood pressure, diastolic blood pressure, and pulse rate were also slightly higher in the non-BSS group, there was no statistically significant difference (Table 1).

### 3.2. Blood Stasis Scores of BSS and Non-BSS Groups within the Metabolic Syndrome

The blood stasis score was calculated by combining the 15 clinical index scores (Table 2). The total blood stasis score was 32, calculated with 3 items with a clinical index of 3 points for a subtotal of 15, 7 items with a clinical index of 2 points for a subtotal of 12, and 3 items with a clinical index of 1 point for a subtotal of 3. The blood stasis score of the BSS group within the metabolic syndrome was 14.09±6.14, higher than that of the non-BSS group with 9.09±5.60 by an average of 5 points (p<0.0001).

**Figure 2:**
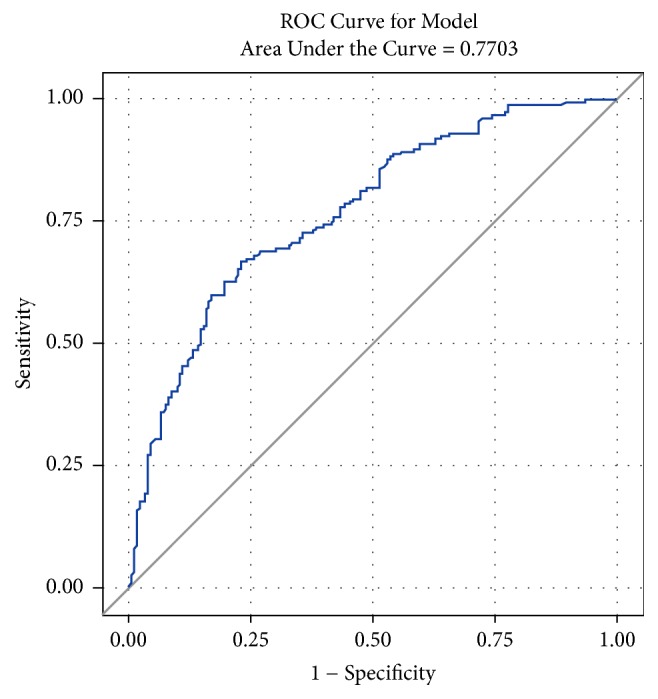
Receiver operating characteristic (ROC) curve for model using logistic regression.

The cut-off value for blood stasis diagnosis using blood stasis score within the metabolic syndrome was 9 points. The diagnostic accuracy using the cut-off value had a sensitivity and specificity of 82.89% and 49.73%, respectively, while the prediction accuracy had a sensitivity and specificity higher than 72.19% and 71.58%, respectively (Table 3). The area under the curve (AUC) of the receiver operating characteristic graph was shown to be approximately 77% (Figure 2).

Logistic regression results revealed that the significant clinical indicators were ‘stabbing pain,' ‘pain at night,' ‘cheek pain,' and ‘easy bruising.' The Cronbach's alpha value for the 15 clinical indicators was 0.70, indicating internal consistency.

## 4. Discussion and Conclusion

Metabolic syndrome is one of the representative chronic illnesses. It involves multiple symptoms such as high blood pressure, abdominal obesity, dyslipidemia, and fasting glucose disorders [13]. There is currently a lack of a BSQ-MS developed with Oriental Medicine clinical indicators. In our study, we used clinical data collected using the previously established BSQ developed in 2013 to evaluate the clinical significance of blood stasis in metabolic syndrome, and evaluated the validity and reliability of blood stasis and non-blood stasis classifications in metabolic syndrome.

The BSQ developed in 2013 collects clinical data for all diseases regardless of the disease. Clinical data centering on the goal diseases, cardiovascular, gynecological, and musculoskeletal disorders, were collected in 2014. In the present study, we utilized the clinical data collected using this questionnaire to analyze clinical data that meets the international standards of metabolic syndrome, in order to utilize them as the basis for developing a BSQ-MS.

Blood stasis diagnostic standards were presented for various diseases such as cerebrovascular diseases, eye conditions, skin diseases, stroke, and pediatric and coronary heart disease between 1998 and 2012 in China [14–20]. In our study, we developed and evaluated a BSQ-MS using the clinical indicators of the BSQ developed in China for coronary heart disease, which has a high relevance to metabolic disease.

The internal consistency of clinical indicators for the diagnosis of blood stasis and non-blood stasis for 370 subjects categorized with metabolic syndrome was adequate at a Cronbach's alpha value of 0.70. Using the blood stasis score calculated as the combination of clinical indicators, we found that the cut-off value that can diagnose blood stasis and non-blood stasis was 9 points. While using the cut-off value accurately diagnoses a blood stasis patient at approximately 83%, the accurate diagnosis of non-blood stasis patients was much at a lower rate of 50%.

Logistic regression showed that accurate prediction of blood stasis in patients diagnosed with blood stasis within metabolic syndrome was 72% and that accurate prediction of non-blood stasis in patients not diagnosed with blood stasis was 72%. The AUC of the receiver operating characteristic graph showed 77%.

In our study, we added the clinical indicators of ‘cheek pain,' ‘blackish red gingiva,' ‘dark purple of palate mucosa,' and ‘easy bruising,' which are related to metabolic disease, to the BSQ developed in Korea based on the blood stasis clinical indicators of coronary heart disease in China. We then compared the sensitivity and specificity of the cut-off value, diagnostic accuracy and prediction accuracy, significant clinical indicators from the logistic regression results, AUC values, and internal consistency (Table 4). The blood stasis cut-off value for the 12 blood stasis clinical indicators from Fu et al. [11], excluding cyanosis, was shown to be 8 points. The sensitivity and specificity of the diagnostic accuracy were 80.43% and 50.28%, respectively, while the sensitivity and specificity of the prediction accuracy were 67.93% and 73.18%, respectively. The Cronbach's alpha value was 0.60, which is lower than the internal consistency standard of 0.70. With the addition of 3 clinical indicators related to metabolic syndrome, the cut-off value for blood stasis score was 7–9 points each. There was not a large difference in sensitivity or specificity with the addition of these clinical indicators. However, with a total of 15 clinical indicators, the cut-off value for the blood stasis score was 9, and sensitivity and specificity in relation to the cut-off value were 82.07% and 46.37%, respectively. The sensitivity and specificity in relation to the predictability from the logistic regression were 72.19% and 71.58%, respectively. Cronbach's alpha value was 0.70, indicating an adequate internal consistency.

Blood stasis and metabolic syndrome are among the representative chronic illnesses. The selection of clinical indicators in BSQ-MS is due to the fact that the clinical indicators presented by Fu et al. (2012) are highly correlated and very similar to blood stasis. In addition, it is possible to classify metabolic diseases as limited (no information for waist circumference) in current blood stasis clinical data.

Although the specificity of diagnosis from the cut-off value did not exceed 50%, and the sensitivity and specificity of predictability from the logistic regression were 72%, in the present study, previous clinical data were used as the basis of developing the metabolic disease questionnaire. Our study reanalyzed previously collected clinical data, rather than collecting data from a new clinical study, and was conducted to develop a BSQ-MS. For the development of the Blood Stasis Questionnaire, we evaluated clinical indicators using metabolic syndrome characteristics. We demonstrated reliability and validity of the BSQ-MS. This study is important because it developed a new questionnaire using previous research data, instead of utilizing clinical data collected via a clinical study conducted on patients with metabolic syndrome. We propose that future studies can be based on the reanalysis of previously collected clinical data from a clinical research, without having to conduct a new clinical research.

## Figures and Tables

**Table 1:** General subject characteristics.

	Total (N=370)	BSS (N=187)	Non-BSS (N=183)	p-value
Sex				0.5467
Male	208 (56.22)	108 (57.75)	100 (54.64)	
Female	162 (43.78)	79 (42.25)	83 (45.36)	
Age (year)	51.06±9.77	49.97±9.59	52.16±9.85	0.0313
BMI (kg/m^2^)				0.2605
Low (≤20)	4 (1.08)	3 (1.60)	1 (0.55)	
Normal (20–24)	172 (46.49)	95 (50.80)	77 (42.08)	
Overweight (25–30)	164 (44.32)	75 (40.11)	89 (48.63)	
Obesity (≥30)	30 (8.11)	14 (7.49)	16 (8.74)	
SBP (mmHg)	131.65±14.47	130.89±14.50	132.43±14.43	0.3070
DBP (mmHg)	82.06±10.06	81.57±9.92	82.56±10.21	0.3472
Pulse rate (BPM)	76.62±10.46	76.26±10.45	76.98±10.48	0.5109

BSS: blood stasis, BMI: body mass index, DBP: diastolic blood pressure, Non-BSS: non-blood stasis, SBP: systolic blood pressure.

P-values were calculated by independent two-sample t-test in continuous variables and chi-squared test in categorical variables.

**Table 2:** Comparison of means for 14 items of the Blood Stasis Syndrome Questionnaire.

	Total (N=370)	BSS (N=187)	Non-BSS (N=183)	p-value
1. Angina pectoris	0.35±0.96	0.35±0.97	0.34±0.96	0.9311
2. Chest pain without angina pectoris	0.64±1.23	0.75±1.30	0.52±1.14	0.0730
3. Blackish red tongue	1.48±1.50	1.70±1.49	1.26±1.49	**0.0049**
4. Ecchymosis of tongue	0.39±1.01	0.55±1.16	0.23±0.80	**0.0025**
5. Stabbing pain	1.44±1.50	1.84±1.46	1.03±1.43	**<0.0001**
6. Pain at night	0.69±0.95	0.97±1.00	0.39±0.80	**<0.0001**
7. Blackish red lips	1.18±0.99	1.32±0.95	1.04±1.00	**0.0066**
8. Sublingual Varicosities	1.25±0.97	1.42±0.91	1.08±1.00	**0.0007**
9. Cheek pain	1.05±1.00	1.32±0.95	0.79±0.98	**<0.0001**
10. Blackish red gingiva	0.68±0.95	0.77±0.98	0.59±0.91	0.0683
11. Dark purple of palate mucosa	0.76±0.97	0.93±1.00	0.58±0.91	**0.0005**
12. Scaly and rough skin	0.35±0.48	0.44±0.50	0.26±0.44	**0.0001**
13. Dark coloration of periocular region	0.54±0.50	0.64±0.48	0.43±0.50	**<0.0001**
14. A dark coloration of the face	0.40±0.49	0.48±0.50	0.32±0.47	**0.0012**
15. Easy bruising	0.76±0.97	0.95±1.00	0.57±0.90	**0.0001**
Total	11.62±6.38	14.09±6.14	9.09±5.60	**<.0001**

p-values were calculated by independent two-sample t-test, and bold values are statistically significant at p<0.05.

BSS: blood stasis, Non-BSS: non-blood stasis.

**Table 3:** Expert physician diagnoses using the blood stasis score cut-off value and prediction by classification of logistic regression.

			Results of classification		
			BSS	Non-BSS	Total
Expert physician results	Diagnostic accuracy	BSS	150 (80.21)	37 (19.79)	187
		Non-BSS	88 (48.09)	95 (51.91)	183
		Total	238	132	370
	Prediction accuracy	BSS	135 (72.19)	52(27.81)	187
		Non-BSS	52 (28.42)	131(71.58)	183
		Total	181	189	370

BSS: blood stasis, Non-BSS: non-blood stasis.

**Table 4:** Additional metabolic syndrome items based on coronary heart disease in China.

			*Diagnostic accuracy*		*Prediction accuracy*					
Signs and symptoms	No. of items	Cut-off value	Sensitivity	Specificity	Sensitivity	Specificity	Significant items by logistic regression	AUC	Cronbach's *α*	Remarks
Fu et al. (2012)	11	8	148 80.43	90 50.28	125 67.93	131 73.18	Stabbing pain, pain at night, sublingual varicosities	75.25%	0.60	Exclusive acrocyanosis

(i) Cheek pain	12	7	155 84.24	80 44.69	125 67.93	127 70.95	Stabbing pain, pain at night, cheek pain	76.07%	0.66	Adding

(i) Blackish red lips	13	8	150 81.52	88 49.16	124 67.39	127 70.95	Stabbing pain, pain at night, cheek pain	76.13%	0.69	Adding

(i) Dark purple of palate mucosa	14	8	155 84.24	84 46.93	126 68.48	129 72.07	Stabbing pain, pain at night, cheek pain	76.32%	0.71	Adding

(i) Cheek pain (ii) Blackish red lips (iii) Dark purple of palate mucosa (iv) Tending to bruise easily	15	9	155 82.89	91 49.73	135 72.19	131 71.58	Stabbing pain, pain at night, cheek pain, tends to bruise easily	77.07%	0.70	Adding

## Data Availability

The data used to support the findings of this study are included within the article.
